# Using machine learning to determine a functional classifier of reward responsiveness and its association with adolescent psychiatric symptomatology

**DOI:** 10.1017/S003329172400240X

**Published:** 2024-11

**Authors:** Robert James Richard Blair, Johannah Bashford-Largo, Ahria Dominguez, Matthew Dobbertin, Karina S. Blair, Sahil Bajaj

**Affiliations:** 1 Child and Adolescent Mental Health Center, Copenhagen University Hospital – Mental Health Services CPH, Copenhagen, Denmark; 2 Department of Clinical Medicine, Faculty of Health and Medical Sciences, University of Copenhagen, Copenhagen, Denmark; 3 Child and Family Translational Research Center, Boys Town National Research Hospital, Boys Town, NE, USA; 4 Center for Brain, Biology, and Behavior, University of Nebraska-Lincoln, Lincoln, NE, USA; 5 Clinical Health, Emotion, and Neuroscience (CHEN) Laboratory, Department of Neurological Sciences, College of Medicine, University of Nebraska Medical Center (UNMC), Omaha, NE, USA; 6 Child and Adolescent Psychiatric Inpatient Center, Boys Town National Research Hospital, Boys Town, NE, USA; 7 Department of Cancer Systems Imaging, Division of Diagnostic Imaging, The University of Texas MD Anderson Cancer Center, Houston, TX, USA

**Keywords:** attention deficit hyperactivity disorder, conduct disorder, major depressive disorder, reward

## Abstract

**Background:**

Machine learning (ML) has developed classifiers differentiating patient groups despite concerns regarding diagnostic reliability. An alternative strategy, used here, is to develop a functional classifier (hyperplane) (e.g. distinguishing the neural responses to received reward *v.* received punishment in typically developing (TD) adolescents) and then determine the functional integrity of the response (reward response distance from the hyperplane) in adolescents with externalizing and internalizing conditions and its associations with symptom clusters.

**Methods:**

Two hundred and ninety nine adolescents (mean age = 15.07 ± 2.30 years, 117 females) were divided into three groups: a training sample of TD adolescents where the Support Vector Machine (SVM) algorithm was applied (*N* = 65; 32 females), and two test groups– an independent sample of TD adolescents (*N* = 39; 14 females) and adolescents with a psychiatric diagnosis (major depressive disorder (MDD), generalized anxiety disorder (GAD), attention deficit hyperactivity disorder (ADHD) & conduct disorder (CD); *N* = 195, 71 females).

**Results:**

SVM ML analysis identified a hyperplane with accuracy = 80.77%, sensitivity = 78.38% and specificity = 88.99% that implicated feature neural regions associated with reward *v.* punishment (e.g. nucleus accumbens *v.* anterior insula cortices). Adolescents with externalizing diagnoses were significantly less likely to show a normative and significantly more likely to show a deficient reward response than the TD samples. Deficient reward response was associated with elevated CD, MDD, and ADHD symptoms.

**Conclusions:**

Distinguishing the response to reward relative to punishment in TD adolescents via ML indicated notable disruptions in this response in patients with CD and ADHD and associations between reward responsiveness and CD, MDD, and ADHD symptom severity.

## Introduction

The assessment of psychiatric patients is a complex process. The Diagnostic and Statistical Manual of Mental Disorders (DSM)-International Classification of Diseases (ICD) approach has improved standardization. However, DSM and ICD have been challenged with respect to their validity/reliability, the process by which the categories are derived and the lack of neurobiological underpinnings to these diagnoses (Cuthbert & Insel, 2013; Insel, 2014). This has fueled demands for the development of neurobiology-based biomarkers relevant to psychiatric disorders and the Research Domain Criteria (RDoC) (Cuthbert & Insel, 2013; Insel, 2014). A number of studies have used machine learning (ML), with various degrees of success, in attempts to determine biomarkers that distinguish patient populations from comparison populations (Dinga et al., 2019; Drysdale et al., 2017; Yang et al., 2018). However, these studies have typically used psychiatric categories as the target for the biomarker. Yet, there are concerns with the reliability of many psychiatric diagnoses (Regier et al., 2013). As such, the RDoC approach suggests an alternative strategy – a focus on dimensions of neuro-cognitive function and a determination of the extent to which perturbations of such function are associated with groups of psychiatric symptoms.

One promising neuro-cognitive function is reinforcement processing. Atypical reinforcement (specifically reward) processing has been linked to a variety of developmental psychiatric diagnoses; e.g., conduct disorder (CD) (Zhang et al., 2023), attention deficit hyperactivity disorder (ADHD) (Grimm et al., 2021), substance use disorders (SUD) (Hubbard et al., 2023), major depressive disorder (MDD) (Stringaris et al., 2015) and generalized anxiety disorder (GAD) (Bashford-Largo et al., 2021). Moreover, an environmental risk variable for the development of these disorders, exposure to maltreatment, has also been associated with disrupted reinforcement processing (Birn, Roeber, & Pollak, 2017; Blair et al., 2021; Gerin et al., 2017). Reinforcement processing involves a number of subcortical and cortical structures, most notably dorsal and particularly ventral striatum, medial frontal and anterior insular cortex (Averbeck & O'Doherty, 2022; Clithero & Rangel, 2014). In brief, dorsal and ventral striatum and ventromedial frontal cortex are particularly responsive to reward information while anterior insular cortex can particularly be seen in response to punishment, particularly if that punishment prompts a future change in response (Averbeck & O'Doherty, 2022; Clithero & Rangel, 2014; Gueguen et al., 2021). Reduced reward responsiveness, reported in the above psychiatric conditions, potentially relates to some of the decision-making difficulties seen in patients with these conditions (Bashford-Largo et al., 2021; Grimm et al., 2021; Stringaris et al., 2015; Zhang et al., 2023). Indeed, previous studies with the task to be used here have shown reduced reward responsiveness in regions including striatum and ventromedial frontal cortex in patients with CD and in patients with GAD (Bashford-Largo et al., 2021; Zhang et al., 2023).

Relating to the complexity of the diagnostic process, there is high comorbidity of the disorders associated with atypical reward processing (i.e. CD, ADHD, MDD, and GAD) (Copeland, Shanahan, Erkanli, Costello, & Angold, 2013; Lundberg et al., 2022). As such, it is unclear if atypical reinforcement processing is associated with the development of all of these disorders. Atypical reward processing may only be associated with the development of certain disorders but because of high co-morbidity many individuals with another disorder may also present with that form of dysfunction. Relatedly, there is the concern with standard univariate data analysis of what has been termed ‘blobology’ (e.g. Hanson et al., 2022); i.e., the identification of a cluster of voxels showing a significant effect (e.g. greater activity in Condition X *v.* Condition Y) as a neural localization of function in the knowledge that an immediate replication will likely identify a (ideally) proximal cluster that will, at best, only partially overlap with the first. This makes determining replicability difficult. For example, it is difficult to determine whether the failure in reward response reported in patients with CD, ADHD, MDD and GAD (Bashford-Largo et al., 2021; Grimm et al., 2021; Hubbard et al., 2023; Stringaris et al., 2015; Zhang et al., 2023) reflect replications of the same dysfunction across patients with different disorders or different forms of dysfunction.

In this study, we determine a Support Vector Machine (SVM)-derived classifier for distinguishing the response to reward relative to the response to punishment in a sample of typically developing (TD) adolescents. Our first goal was to use this classifier to determine the extent to which patients with disorders associated with reduced reward responding would be categorized within the group showing a significantly weak reward response (i.e. a reduced distance of their reward response from the hyperplane; for greater details on our theoretical underpinnings, see Supplemental Material SM1). Our second goal was to determine the extent to which reward response categorization was associated with specific forms of symptom. Based on the previous literature (Averbeck & O'Doherty, 2022; Clithero & Rangel, 2014; Gueguen et al., 2021), it was predicted that (i) a hyperplane of high accuracy would be identified that could distinguish the response to reward from that to punishment; and (ii) this hyperplane would include features associated with regions including ventromedial prefrontal cortex and striatum (reward) and anterior insular cortex (punishment). Further, based on clinical neuroscience work (Bashford-Largo et al., 2021; Grimm et al., 2021; Stringaris et al., 2015; Zhang et al., 2023), it was predicted that (iii) patients with CD, ADHD, MDD, and/or GAD would show over-representation within the category of ‘significantly weak reward response’ (see SM1; cf. Bashford-Largo et al., 2021; Grimm et al., 2021; Hubbard et al., 2023; Stringaris et al., 2015; Zhang et al., 2023); and (iv) an individual's distance from the hyperplane (DFH) for reward responses would be inversely associated with severity of symptoms associated with CD, ADHD, MDD, and/or GAD.

## Methods

### Participants

The current study included data collected from 299 youths between 10 and 19 years of age (mean age = 15.07 ± 2.30 years, 117 females). Some of these data have been previously reported using more conventional group-level univariate approaches (cf. Bashford-Largo et al., 2021; Zhang et al., 2023). Participants were recruited from a residential care facility and the surrounding community. Individuals recruited from the residential facility had been referred for severe behavioral and mental health problems. Participants from the community were recruited through flyers or social media. There were three groups of adolescents: TD adolescents on whose data the SVM ML was conducted (*N* = 65; 32 females), a randomly chosen sample of TD adolescents independent of the first group (*N* = 39; 14 females), and adolescents with a psychiatric diagnosis (*N* = 195; 71 females); for full participant characteristics, see Table 1. Clinical characterization of all participants was done through psychiatric interviews by licensed and board-certified child and adolescent psychiatrists with the participants and their parents to adhere closely to common clinical practice. Institutional Review Board approval was acquired before data collection began. For details on exclusion criteria and consent procedure, see Supplemental Material 2.

**Table 1.** Participant characteristics of the three participant groups

	TD_Train_ (*N* = 65)		TD_Test_ (*N* = 39)		Patients (*N* = 195)		TD_Train_ v TD_Test_		TD_Test_ v Patients	
	Mean	s.d.	Mean	s.d.	Mean	s.d.	*F* =	*p* =	*F* =	*p* =
Age	14.20	2.46	13.92	2.70	15.57	2.00	0.30	0.585	**20.08**	<0.001
IQ	106.86	12.51	109.26	13.73	102.09	12.21	0.83	0.364	**10.74**	0.001
Family income	114 021	48 949	124 800	54 093	76 344	56 114	0.30	0.585	**12.23**	<0.001
	*N*	%	*N*	%	*N*	%	χ^2^	*p* =	χ^2^	*p* =
Sex (N female)	32	49.23	14	35.90	71	36.41	1.76	0.185	0.004	0.952
Handedness (right-handed)	59	90.77	32	82.05	178	91.28	1.62	0.204	2.92	0.087
CD	0	–	0	–	105	53.85	–	–	–	–
ADHD	0	–	0	–	140	71.79	–	–	–	–
MDD	0	–	0	–	35	17.95	–	–	–	–
GAD	0	–	0	–	63	32.31	–	–	–	–
Antipsychotic	0	–	0	–	21	10.77	–	–	–	–
Stimulant	0	–	0	–	45	23.08	–	–	–	–
SSRI	0	–	0	–	39	20.00	–	–	–	–
	Mean	s.d.	Mean	s.d.	Mean	s.d.	*F* =	*p* =	*F* =	*p* =
SDQ-CP	0.28	0.62	0.54	1.12	4.70	2.94	2.09	0.152	**67.24**	<0.001
RPS-Reactive	4.87	2.08	4.91	1.69	9.14	3.63	0.01	0.923	**43.17**	<0.001
RPS-Proactive	3.31	0.82	3.29	0.72	5.96	3.19	0.02	0.904	**23.19**	<0.001
Conners ADHD	0.22	0.77	0.26	0.98	8.49	6.20	0.04	0.846	**61.08**	<0.001
SDQ-Hyperactivity	1.09	1.09	1.28	2.02	6.35	2.65	0.346	0.558	**115.34**	<0.001
SDQ-Emotional	0.93	1.24	0.67	0.79	4.20	2.88	1.29	0.259	**53.05**	<0.001
CDI	3.61	3.93	2.70	2.44	10.95	8.57	1.58	0.212	**33.55**	<0.001
SCARED Total	12.52	8.13	9.11	6.12	20.07	15.91	4.95	0.028	**17.45**	<0.001
SCARED GAD	3.98	3.26	2.50	2.43	6.29	5.01	5.87	0.017	**20.71**	<0.001
Abuse	16.80	3.93	16.13	1.99	23.03	10.03	0.97	0.326	**18.25**	<0.001
Neglect	11.55	2.46	11.38	1.89	16.92	7.05	0.13	0.724	**23.65**	<0.001
AUDIT	0.17	0.63	0.13	0.48	2.96	5.49	0.10	0.749	**10.04**	0.002
CUDIT	0.32	1.36	0.00	–	7.42	9.16	2.14	0.147	**24.81**	<0.001
ICU	15.20	5.73	14.87	6.81	23.73	8.47	0.07	0.793	**37.69**	<0.001
ARI	0.86	1.06	0.74	0.99	3.35	3.17	0.90	0.344	**25.80**	<0.001

TD, Typically developing; CD, conduct disorder; ADHD, attention deficit hyperactivity disorder; MDD, major depressive disorder; GAD, generalized anxiety disorder; SDQ-CP, Strengths and Difficulties Questionnaire – Conduct Problems subscale; RPS-Reactive, Reactive Proactive Scale – Reactive subscale; RPS-Proactive, Reactive Proactive Scale – Proactive subscale; Conners ADHD, Conners 3 ADHD Index; SDQ-Hyperactivity, Strengths and Difficulties Questionnaire – Hyperactivity subscale; SDQ-Emotional, Strengths and Difficulties Questionnaire – Emotional Problems subscale; CDI, Child Depression Inventory; SCARED Total, Screen for Child Anxiety Related Emotional Disorder Total score; SCARED GAD, Screen for Child Anxiety Related Emotional Disorder GAD subscale; Abuse, Childhood Trauma Questionnaire abuse score (Sexual + Physical + Emotional abuse); Neglect, Childhood Trauma Questionnaire abuse score (Physical + Emotional neglect); AUDIT, Alcohol Use Disorder Identification Test; CUDIT, Cannabis Use Disorder Identification Test; ICU, Inventory of Callous Unemotional Traits; ARI, Affective Reactivity Index.

### Measures

For details on the **clinical measures** see Supplemental Material 3.

**Passive avoidance learning (PAL) task:** The PAL task, used extensively in previous work with adolescents (Bashford-Largo et al., 2021; White et al., 2013), is a probabilistic instrumental learning paradigm that presents participants with cues that, if acted upon, offer a chance to win or lose virtual money (see for further details Supplemental Material 3 and Supplemental Figure 1). In each trial, one of four cue shapes was presented. Participants chose whether to respond to the cue. If they chose to respond, a feedback screen was presented for 1500 ms informing them that their choice resulted in winning or losing money. Feedback followed a probabilistic reinforcement schedule. Cues were presented in a random order. Each of the four cues was presented 27 times (108 total trials).

For details on **fMRI parameters**, **fMRI data processing, individual level analysis and movement data**, see Supplemental Material 4.

### Data analysis

#### Demographic and clinical data

Demographic (age, IQ and sex ratios) and clinical (self-report assessments) scores are presented for: (i) the whole sample; (ii) the 65 TD *training* participants on whom the SVM ML was conducted; (iii) an independent sample of 39 TD *test* adolescents randomly chosen to be an independent sample; and (iv) the 195 *clinical* participants.

#### Feature creation

The Schaefer's Atlas (Schaefer et al., 2018) was used to parcellate the whole brain into 400 regional parcellations (for additional details, see Supplemental Material 5). Subject-wise and hemispheric-wise BOLD response to the receipt of reward and punishment information was determined for each of the 400 regions as well as 12 bilateral subcortical regions (the thalamus, caudate, putamen, nucleus accumbens, hippocampus and amygdala). The whole-brain parcellation into 400 cortical and 12 subcortical regions was performed using the *mri_surf2surf*, *mris_anatomical_stats*, and *aparcstats2table* pipelines following the FreeSurfer *recon-all* pipeline (Fischl et al., 2002)

#### Feature selection and ML analysis

For details on **Feature selection and ML analysis**, see Supplemental Material 5.

### Associations between distance from the hyperplane and demographic and clinical data

Exploratory correlation analyses were conducted across the whole sample to provide information on associations between DFH for reward and demographic and clinical data.

### Normed reward DFH categories and clinical associations

To determine populations of participants who showed typical/atypical responses to rewards, the DFH data for the independent test sample of 39 randomly selected TD adolescents and the 195 clinical participants was normed on the basis of the mean and standard deviation (s.d.) DFH data of the training sample of 65 TD adolescents. Participants were then categorized according to whether they showed responses to reward that were >2 s.d., 1 to 2 s.d., 1 to −1 s.d., −1 to −2 s.d. and <-2 training s.d. from the training sample mean. Subsequently, χ^2^ analyses were conducted to determine whether proportions of participants in these five categorizations differed between the training and test TD samples, and the training TD sample and the clinical adolescent participants. Chi-square analyses were also conducted to determine whether proportions of participants in these five categories differed between the TD samples and the training and adolescents presenting with psychiatric diagnoses (CD, MDD, ADHD, and GAD).

Following this, *t* tests were conducted to determine whether there were significant differences in symptom severity between participants showing *typical* responses to reward (i.e. with a DFH for reward trials from the hyperplane that was 1 to −1 training sample s.d. from the training sample mean) and those showing significant *reduced* responses to reward (i.e. with DFH for reward trials from the hyperplane that was −2 training sample s.d. from the training sample mean).

## Results

### Demographic and clinical data

Table 1 reports the demographic and clinical data for: (i) the 65 TD participants on whom the SVM ML was conducted (TD_Train_); (ii) an independent sample of 39 randomly chosen TD adolescents to be an independent sample (TD_Test_); and (iii) the 195 clinical participants (CP). While the TD_Train_ and TD_Test_ samples were comparable, unsurprisingly both groups of TD adolescents showed significant differences in many of these indices relative to the clinical participants (see Table 1). Supplementary Table 1 reports the number of patients with each comorbidity pattern.

### Generalized model performance and feature identification

With respect to the BOLD response data for response to reward *v.* punishment, our SVM ML analysis identified a hyperplane with accuracy = 80.77%, sensitivity = 78.38% and specificity = 88.99%.

**Figure 1.**
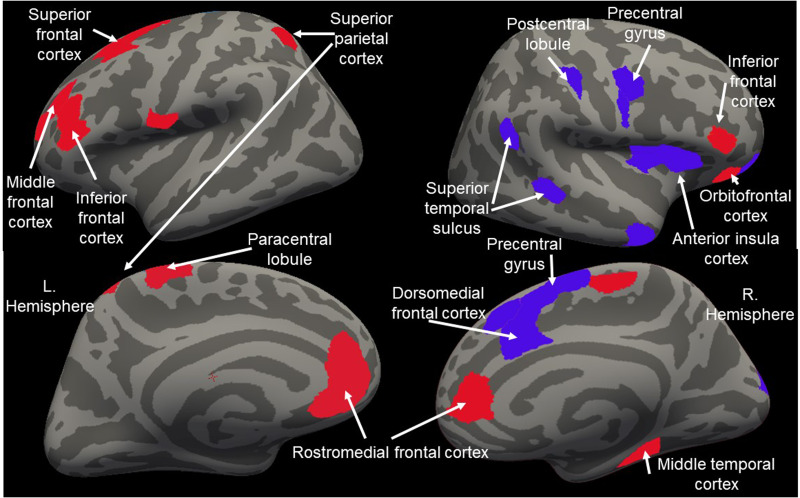
The features selected from the TD Training sample. The 39 features, identified via LASSO (see Supplemental Materials) differentiating the BOLD response to the receipt of reward *v.* punishment and their functional roles. Regions in red showed greater responses to reward. Regions in blue showed greater responses to punishment.

A total of 39 features were identified (see Fig. 1). Twenty of these features (including rostro- and ventro-medial and orbital frontal cortex, putamen and nucleus accumbens) showed significantly greater responses to reward relative to punishment while 14 (including dorsomedial frontal and anterior insular cortices) showed significantly greater responses to punishment relative to reward (see Fig. 1).

### Associations between DFH and demographic and clinical data

With respect to demographic variables, correlational analyses (age, IQ) and one-way ANOVAs (sex) revealed no significant associations between these variables and DFH for responses to received reward for either the test samples (TD_Test_ & CP; Table 2). For received punishment, this was the same except that punishment DFH was inversely related to IQ (see Table 2 and Supplemental Table 2).

**Table 2.** Associations between distance from the hyperplane and demographic data (Pearson's *R* values)

	Reward	Punishment
Age	−0.01	−0.06
IQ	−0.10	−0.14*
SDQ-CP	−0.17*	−0.05
RPAQ-Reac	−0.22**	−0.07
RPAQ-Proactive	−0.15	0.01
Conners ADHD	−0.17*	−0.09
SDQ-Hyperactivity	−0.14	−0.03
SDQ-Emotional	−0.17*	−0.04
CDI	−0.13	−0.13
SCARED Total	−0.02	−0.12
SCARED GAD	0.02	−0.13
Abuse	0.04	−0.10
Neglect	−0.16*	−0.11
AUDIT	−0.03	0.03
CUDIT	−0.06	0.03
ICU	−0.15*	−0.04
ARI	−0.03	0.03

SDQ-CP, Strengths and Difficulties Questionnaire – Conduct Problems subscale; RPAQ-Reactive, Reactive Proactive Aggression Scale – Reactive subscale; RPAQ-Proactive, Reactive Proactive Aggression Scale – Proactive subscale; Conners ADHD, Conners 3 ADHD Index; SDQ-Hyperactivity, Strengths and Difficulties Questionnaire – Hyperactivity subscale; SDQ-Emotional, Strengths and Difficulties Questionnaire – Emotional Problems subscale; CDI, Child Depression Inventory; SCARED Total, Screen for Child Anxiety Related Emotional Disorder Total score; SCARED GAD, Screen for Child Anxiety Related Emotional Disorder GAD subscale; Abuse, Childhood Trauma Questionnaire abuse score (Sexual + Physical + Emotional abuse); Neglect, Childhood Trauma Questionnaire abuse score (Physical + Emotional neglect); AUDIT, Alcohol Use Disorder Identification Test; CUDIT, Cannabis Use Disorder Identification Test; ICU, Inventory of Callous Unemotional Traits; ARI, Affective Reactivity Index.

*p < 0.05; ** *p* < 0.01; *** *p* < 0.005.

With respect to clinical variables, correlational analyses revealed significant associations between DFH for responses to reward and clinical measures indexing conduct problems, hyperactivity, depression and neglect across the test sample – though none of these were significant for DFH for responses to punishment (see Table 2).

### Normed reward DFH categories and clinical associations

**Figure 2.**
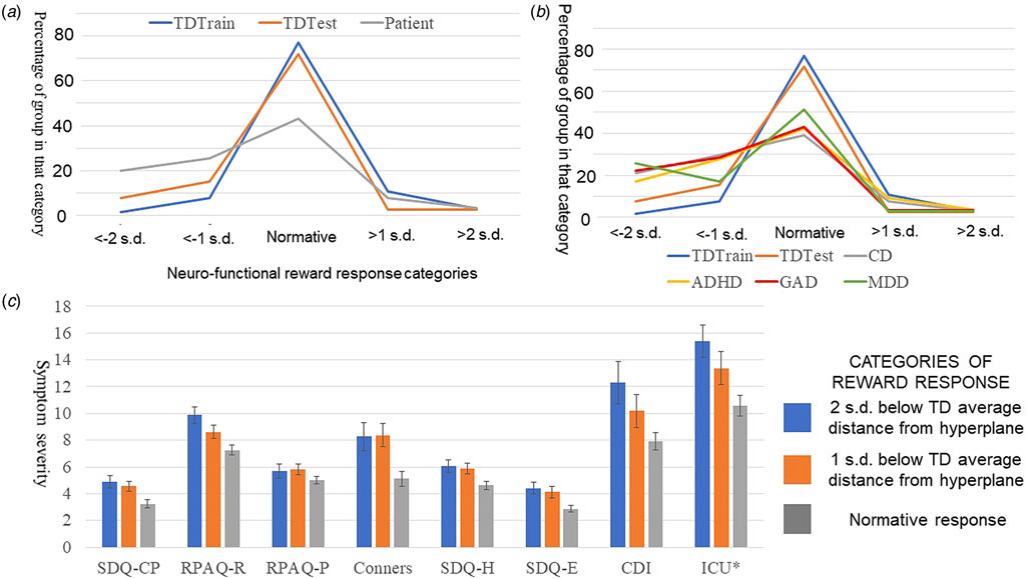
Normed reward DFH categories and clinical associations. (a) The proportions of each of the three participant groups in each category of normed response to reward. While the proportions of participants in the normed response to reward groups between the training and test TD samples did not significantly differ, there were marked differences with the patient population; (b) The proportions of the two TD groups (training and test) and the patients broken down by diagnosis; (c) The significant association between category of reward response groups and specific symptom groups. TD, Typically developing; GP, Group; s.d., standard deviation; SDQ-CP, Strengths and Difficulties Questionnaire – Conduct Problems subscale; RPAQ-R, Reactive Proactive Aggression Questionnaire – Reactive subscale; RPAQ-P, Reactive Proactive Aggression Questionnaire – Proactive subscale; Conners, Conners 3 ADHD Index; ADHD, attention deficit hyperactivity disorder; SDQ-H, Strengths and Difficulties Questionnaire – Hyperactivity subscale; SDQ-E, Strengths and Difficulties Questionnaire – Emotional Problems subscale; CDI, Child Depression Inventory; ICU, Inventory of Callous Unemotional Traits (*note all ICU scores have had 10 subtracted to fit easier within the figure). *Note*: RPAQ-P results are presented for comparison purposes. They were not significant (see Table 3).

Figure 2a depicts the proportions of each of the three participant groups in each category of normed response to reward. Chi-square analyses revealed highly significant differences in the proportions of participants in the normed response to reward groups between the patient group and TD_test_ groups (χ^2^(df[4], *N* = 234) = 11.12, *p* = 0.025); for the number of participants within each normed reward DFH category, see Supplemental Table 3. Two features of this result should be noted:

**Table 3.** Associations of level of reward response (distance from hyperplane) groups and symptom severity (TD_Test_ and participants with diagnoses)

	2 sd below TD_Train_ average distance from hyperplane		1 sd below TD_Train_ average distance from hyperplane		Normative (>1 sd below and <1 sd above TD_Train_ average distance from hyperplane)			
	Mean	Std. deviation	Mean	Std. deviation	Mean	Std. deviation	*F*	*p*
SDQ-CP	4.91	3.04	4.55	2.79	3.23	3.24	4.93	0.008
RPAQ-Reactive	9.89	3.98	8.62	3.64	7.26	3.73	5.24	0.007
RPAQ-Proactive	5.71	3.33	5.82	3.15	5.03	3.04	0.93	0.396
Conners ADHD	8.28	6.81	8.38	6.69	5.11	5.81	5.13	0.007
SDQ-Hyperactivity	6.05	3.01	5.89	3.10	4.62	3.33	3.71	0.027
SDQ-Emotional	4.42	2.86	4.12	3.30	2.85	2.63	5.10	0.007
CDI	12.33	10.27	10.18	9.15	7.91	6.91	4.29	0.015
SCARED Total	18.44	15.66	20.33	16.30	17.37	14.78	0.64	0.526
SCARED GAD	5.44	4.85	6.26	5.17	5.61	4.85	0.42	0.661
Abuse	23.76	10.49	19.46	6.07	22.11	10.49	2.63	0.074
Neglect	19.24	9.37	15.61	5.35	15.07	6.12	6.05	0.003
AUDIT	3.90	5.66	2.12	3.84	1.82	4.57	2.85	0.060
CUDIT	7.74	9.10	7.20	9.33	4.71	8.01	2.38	0.096
ICU	25.40	7.85	23.38	9.12	20.58	8.31	5.60	0.004
ARI	3.00	3.17	3.29	3.14	2.65	2.95	0.85	0.428

SDQ-CP, Strengths and Difficulties Questionnaire – Conduct Problems subscale; RPAQ-Reactive, Reactive Proactive Aggression Questionnaire – Reactive subscale; RPAQ-Proactive, Reactive Proactive Aggression Questionnaire – Proactive subscale; Conners ADHD, Conners 3 ADHD Index; ADHD, attention deficit hyperactivity disorder; SDQ-Hyperactivity, Strengths and Difficulties Questionnaire – Hyperactivity subscale; SDQ-Emotional, Strengths and Difficulties Questionnaire – Emotional Problems subscale; CDI, Child Depression Inventory; SCARED Total, Screen for Child Anxiety Related Emotional Disorder Total score; SCARED GAD, Screen for Child Anxiety Related Emotional Disorder GAD subscale; Abuse, Childhood Trauma Questionnaire abuse score (Sexual + Physical + Emotional abuse); Neglect, Childhood Trauma Questionnaire abuse score (Physical + Emotional neglect); AUDIT, Alcohol Use Disorder Identification Test; CUDIT, Cannabis Use Disorder Identification Test; ICU, Inventory of Callous Unemotional Traits; ARI, Affective Reactivity Index.

First, this result is driven by the proportions of patients in the low reward response groups. Conducting a follow up χ^2^ tests for proportions in the normative (>1sd_Train_ below and <1sd above average Train_DFH_) and low response groups (>1 or >2 sd_Train_ *below* average Train_DFH_) revealed significant differences in proportions between the TD_test_ and patient groups, (χ^2^(df[2], *N* = 210) = 9.22, *p* = 0.01). In contrast, follow up χ^2^ tests for proportions in the normative and high response groups (>1 or >2 sd_TD_ *above* average Train_DFH_) revealed no significant differences in proportions (χ^2^(df[2], *N* = 136) = 3.31, *p* = 0.19); see ST2.

Second, the differences in the proportions of participants in the normed response to reward groups between the TD_test_ and patient groups were significant for CD (χ^2^(df[4], *N* = 144) = 12.63, *p* = 0.013) and ADHD (χ^2^(df[4], *N* = 179) = 11.06, *p* = 0.026) though not for GAD (χ^2^(df[4], *N* = 102) = 8.63, *p* = 0.071) and MDD (χ^2^(df[4], *N* = 74) = 4.97, *p* = 0.290).

It should also be noted that χ^2^ analyses revealed no significant differences in the proportions of participants in the normed response to reward groups between the TD_test_ and TD_Train_ samples, χ^2^(df[4], *N* = 104) = 6.01, *p* = 0.199). Additional analyses contrasting the TD_Train_ and patient groups are presented in Supplemental Material 6.

### Exploratory examination of symptom severity as a function of reward response categorization

Univariate ANOVAs revealed that participants showing significant reduced responses to reward showed significantly greater levels of aggression and conduct problems (as indexed by SDQ-CP, RPAQ reactive aggression and ICU), ADHD (as indexed by both Conners and Strengths and Difficulties Questionnaire [SDQ]-Hyperactivity) and depression/emotional problems (as indexed by both CDI and SDQ-Emotional problems) relative to participants showing typical responses to reward (see Table 3). They also showed significantly greater prior exposure to neglect (though not abuse). However, there were no significant group differences for irritability or anxiety (see Table 3, see also Supplemental Table 4). Figure 2c shows symptom ADHD and depression/emotional problems symptom severity data as a function of level of reward response groups.

### Sensitivity analyses

While reward_DFH_ was not associated with age, IQ or sex, the TD_Test_ and clinical cases differed on these measures. For this reason, sensitivity analyses were conducted controlling for these measures (see Supplemental Material 7).

## Discussion

The goals of the current project were to use an SVM-derived classifier distinguishing the response to reward relative to the response to punishment to determine the extent to which an individual failing to distinguishing the response to reward from the identified hyperplane was associated with psychiatric symptomatology. There were four main findings: First, a hyperplane distinguishing the BOLD response to reward *v.* punishment could be identified with high accuracy, sensitivity and specificity. Second, there were significant associations between reduced reward DFH and psychiatric symptom severity. Third, deriving the mean and s.d. of the directional DFH for the reward response from the data from the training sample of TD adolescents, allowed the directional data from all other participants to be normed. This revealed notable differences in the distribution of norm-based categories in the TD adolescents relative to the adolescents with psychiatric pathology. Fourth, adolescents with *significant* reduced reward DFH (>2 s.d.s DFH from the TD mean DFH toward the hyperplane) showed significant elevated CD, ADHD, SUD and MDD symptomatology and higher levels of past neglect relative to those adolescents showing a normative reward response.

A growing body of ML work with MRI data has focused on differentiating patients from comparison populations (Drysdale et al., 2017; Eslami, Almuqhim, Raiker, & Saeed, 2020; Hao et al., 2023; Yang et al., 2018). While this work is interesting, it is hampered by the relatively low inter-rater reliability of some psychiatric diagnoses (Reed et al., 2018; Regier et al., 2013). Concerns with the diagnostic process detailed in DSM-5 and ICD-11 (Cuthbert & Insel, 2013; Insel, 2014) led to the development of Research Domain Criteria (RDoC). The goal of the RDoC project was to define basic dimensions of functioning (neuro-cognitive mechanisms such as those mediating negative or positive valence) cutting across disorders as traditionally defined (Cuthbert & Insel, 2013; Insel, 2014). The approach developed in the current paper operationalizes an objective ML-based method for identifying neuro-cognitive systems involved in specific task-based functions that can be compromised in patients across diagnostic groups. Importantly, it offers the possibility of individual level assessments of specific neuro-cognitive functions – in this case the response to reward.

A considerable body of replicated work has identified the neural systems responsive to reinforcement information (Averbeck & O'Doherty, 2022; Clithero & Rangel, 2014; Gueguen et al., 2021). Importantly, the current study identified a hyperplane, implicating these neural systems, that distinguished the BOLD response to reward *v.* punishment with high accuracy, sensitivity and specificity. In line with considerable previous work (Averbeck & O'Doherty, 2022; Clithero & Rangel, 2014; Gueguen et al., 2021), striatum and regions of medial frontal cortex were implicated and showed greater responses to reward than punishment. Also, and in line with previous work (Gueguen et al., 2021), a large region of anterior insula cortex was also implicated, identifying greater responses to punishment than reward.

The current paper focused on pathology associated with an atypically reduced response to reward (DFH of the reward response). This was because a deficient response to reward has been consistently linked with a number of pathologies (Bashford-Largo et al., 2021; Grimm et al., 2021; Stringaris et al., 2015; Zhang et al., 2023). In contrast, while there are reports of atypical responses to punishment, particularly in patients with CD and ADHD (White et al., 2013), these findings are far less consistently reported. The current data indicated that an atypical response to reward is seen in a significant proportion of the patients in the current sample whether they received diagnoses of MDD, GAD, ADHD, or CD. Patients with the diagnoses were less likely to show a normative response to reward (reward response DFH) and more likely to show a deficient response to reward (<2 training set s.d.s from the training set mean distance) than typical developing participants. Reward response DFH was negatively correlated with symptom classes associated with these diagnoses (see Table 2). These results are consistent with previous reports associating these disorders with a deficient response to reward (Bashford-Largo et al., 2021; Grimm et al., 2021; Stringaris et al., 2015; Zhang et al., 2023). There are two important points to note: First, adolescents with these disorders were *not* more likely to show an *exaggerated* reward response (>2 TD_Train_ sds from the TD_Train_ mean distance). This is consistent with most previous work where groups of patients with these disorders show reduced reward responding (Blair, Veroude, & Buitelaar, 2018; Grimm et al., 2021; Stringaris et al., 2015). Second, the current data strongly suggests the transdiagnostic nature of the disrupted reward response. Prior work has pointed to, for example, reduced striatal activations in patients with these disorders (Bashford-Largo et al., 2021; Grimm et al., 2021; Hubbard et al., 2023; Stringaris et al., 2015; Zhang et al., 2023). However, the specific regions have differed across studies (both within disorder studies as well across disorder studies), cf. the ‘blobology problem’ (e.g. Hanson et al., 2022). In contrast, the current paper indicates that a reward response classifier, developed from a TD sample and which generalizes to an independent TD sample, identifies significantly increased levels of disrupted recruitment in patients with CD, ADHD, MDD, and GAD.

There are several features to note about these results. First, the correlations of DFH with symptom severity, while significant, were relatively low. This form of neuro-cognitive function is clearly only one amongst others contributing to patients' symptoms. Indeed, all four disorders are also associated with other forms of atypical neuro-cognitive function relating, for example, to negative biases, response control dysfunction and reduced responsiveness to distress cues (e.g. Blair, 2022; Duyser et al., 2022). In this regard, it is notable that less than 30% within each patient group showed significant reduced reward response (i.e. <2 TD_training_ sds from the TD_training_ mean distance). The dysfunction identified appeared to contribute to the patient's pathology (at least with respect to CD, MDD, SUD, and ADHD severity) but is not a complete neurobiological account for all patients for any of these disorders. Perhaps, such forms of dysfunction exist (e.g. all patients with ADHD might have significant response control dysfunction (Barkley, 1997) – though this position is not supported by the neuropsychological data (Coghill, Seth, & Matthews, 2014)). Critically, though reward response dysfunction might be considered a treatment target relevant to significant numbers of patients with these disorders.

Second, the distributions in reward response differed markedly in the TD and clinical participants (see Fig. 2). Patients with a diagnosis of GAD, MDD, ADHD, and CD all showed significantly different distributions in normed response relative to TD adolescents; i.e., a smaller proportion of patients with these diagnoses showed normative responses and a greater proportion showed deficient responses (though there were no significant differences in proportions showing exaggerated responses). This could lead to the conclusion that all four disorders are at least partially underpinned by atypical reward responses (Bashford-Largo et al., 2021; Grimm et al., 2021; Stringaris et al., 2015; Zhang et al., 2023). However, it is worth noting that these disorders are highly comorbid (Copeland et al., 2013). Importantly, examination of symptom severity differences between level of reward response groups suggested that atypically reduced recruitment of the reward response was associated with elevated CD, ADHD, SUD, and MDD symptoms but not GAD symptoms. Notably, the results were echoed for independent measures (i.e. RPAQ and SDQ-CP for CD, SDQ-Emotional problems and CDI for MDD and SDQ-Hyperactivity and Conners ADHD scale for ADHD). As such, it can be argued that atypical reward responses may be associated with pathology in CD, ADHD, and MDD.

Six caveats should be considered with respect to the current results. First, atypical reward responsiveness was associated with both CD/ADHD and MDD symptoms despite the differences in the symptoms of CD & ADHD *v.* MDD. Both have been linked with reduced reward responsiveness using the same or similar tasks (Grimm et al., 2021; Stringaris et al., 2015). However, it must be assumed that how the reduced reward response information is being utilized determines its behavioral manifestation; i.e., a reduced experience of reward might underpin MDD while disrupted decision-making based on poor reinforcement information might underpin impulsivity (Grimm et al., 2021; Stringaris et al., 2015). Other tasks might be better to identify separable functional disruptions in patients with these disorders. Second, we did not implement structured or semi-structured diagnostic interview. As such diagnostic reliability can be challenged (c.f. Regier et al., 2013). Indeed, this problem with diagnosis was a major motivator of the study. It is true that use of structured interviews such as the Kiddie Schedule for Affective Disorders and Schizophrenia improve reliability scores. However, kappas often remain below 0.8 indicating non-optimal reliability (de la Peña et al., 2018). Thus, while we cannot be certain the extent to which reward-related pathology relates to the disorders under study, it appears clear from the symptom severity analyses that CD, ADHD, and MDD might be particularly impacted and that atypical reward responsiveness represents a treatment target that, if addressable, might significantly ameliorate the difficulties of many adolescent psychiatric patients. Third, the current study revealed dysfunction in the integrated response to reward. It did not investigate responses to rewards as a function of expectations based on previous reinforcement history (i.e. prediction error signaling). However, as noted in previous analyses of data from this task (Zhang et al., 2023), pilot analyses of BOLD response data on the current PA task in healthy participants revealed that this task implementation was not optimized to reveal a strong expected value/prediction error signal. Future computational modeling-based work with other tasks will investigate this issue. Fourth, recent work has seriously challenged the use of contrast-based data, rather than individual regressor based analyses, in individual differences research because of data reliability concerns. It is important to note here though: (a) group-based analyses are relatively insensitive to the reliability concerns of contrast-based analyses (see Blair, Mathur, Haines, & Bajaj, 2022; Chen et al., 2021). The determination of the hyperplane was derived from a group-based analysis; and (b) all the individual difference analyses in this study were based on a single condition; reward response DFH (i.e. not a contrast against another condition). Fifth, increasing the size of the training dataset has advantages in ML analyses (e.g. Koppe, Meyer-Lindenberg, & Durstewitz, 2021) and the training set N here (65) could be considered small. However, it is important to note that training set N is partly a function of data complexiety (Koppe et al., 2021) and the critical question is applicability of the obtained hyperplane to independent datasets. As can be seen in Fig. 2, the hyperplane allowed the characterization of the data of both the independent TD sample and even, albeit less successfully, the patients. Sixth, the TD and clinical samples differed significantly in a number of variables (age, IQ, and family income). Sensitivity analyses indicated our main findings were not driven by age or IQ covariates. However, such sensitivity analyses were not possible to conduct with respect to family income (a number of our clinical participant families were not prepared to disclose income data). Notably, there are indications that poverty may be related to reduced reward responsiveness (Palacios-Barrios et al., 2021). Indeed, reduced reward responsiveness appears to mediate the association between poverty and depression in a paper where a pre-specified ROI was used to address the issue (Palacios-Barrios et al., 2021). We hope to address the issue via the integrated functional response identified via the current SVM ML approach in future work.

In conclusion, this study demonstrated that: (i) it is possible to identify a functional signal that differentiates reward from punishment processing in TD adolescents with high accuracy; (ii) a significant proportion of adolescent patients with MDD, GAD, CD, and ADHD show disruption in their response to reward. Critically, this paper demonstrates that reduced response to reward is a transdiagnostic phenomenon (at least to these disorders) as indexed by a classifier of functional integrity developed from TD participants; and (iii) extent of disrupted reward response relates to CD, ADHD, SUD, and MDD symptom levels. Functional classifiers such as that developed in this study could be used as treatment targets and indices of treatment responsiveness. Such classifiers could lead to an individualized approach to the treatment of adolescent psychiatric patients.

## Supporting information

Blair et al. supplementary material Blair et al. supplementary material

## Data Availability

The data cannot be published or shared externally without permission granted by Boys Town National Research Hospital.

## Supplementary material

The supplementary material for this article can be found at https://doi.org/10.1017/S003329172400240X.
