# Supplementary material for: Using machine learning to determine a functional classifier of reward responsiveness and its association with adolescent psychiatric symptomatology
Source: Psychol Med. 2024 Nov 18;54(15):4212–21. doi: 10.1017/S003329172400240X (PMC11650163; doi:10.1017/S003329172400240X)
Supplement: Blair et al. supplementary material [file S003329172400240Xsup001.docx]

**Supplemental Material**

1. **Further details on the theoretical underpinnings of the approach**

*Developing the hyperplane from TD_Train_:* Considerable data indicates that the response to the receipt of reward can be distinguished from that to the receipt of punishment (Averbeck & O'Doherty, 2022, Clithero & Rangel, 2014, Gueguen et al., 2021). As such, it can be hypothesized that SVM can be used to identify a hyperplane distinguishing these responses in typically developing (TD) adolescents.

The SVM aims to find the hyperplane that best distinguishes two classes (here neural responses to reward vs. neural responses to punishment); i.e., the hyperplane that maximizes the distances from the support vectors corresponding to individual participants’ reward and punishment to the hyperplane (see Supplemental Figure SF2a). In SF2a, there are only two features examined (X_1_ and X_2_). However, the SVM conducted on the current data is multidimensional (39 features).

*Distance from hyperplane (DFH):* The DFH can be considered a measure of functional integrity. The greater the DFH, the more the individual’s response to, for example, reward is differentiated from the neural response to punishment. Using the TD_Train_ DFH data it is possible to determine a TD_Train_ mean_DFH_ (the dark blue line in the schematic Supplemental Figure SF2a) and standard deviation_DFH_ for reward (and punishment) trials. This allows all participants’ DFH data to be normed to the TD_Train_ data. In SF2a, 2 sd_DFH_ +/- the mean_DFH_ are depicted. Participants showing, for example, a reward response < +/-2 sd_DFH_ from the mean_DFH_ can be considered to be normative. In the schematic example SF2a, six participants showed a normative reward response (within 2 TD_Train_ sd_DFH_). One participant shows a significantly weaker response (dark orange dot); their reward response distance from the hyperplane is less than 2 TD_Train_ sd_DFH_ from the TD_Train_ mean_DFH_.

*Categorization of participants as a function of their TD_Train_ sd_DFH_ from the TD_Train_ mean_DFH_:* This norming process allows the categorization of participants as a function of their normed DFH based on the *HC_Train_* data (see Supplemental Figure SF2b where the 9 schematic participants from SF2a are depicted). If functional integrity of the system is related to symptom severity then participants in the “weaker” response categories (participants showing reduced response response DFH) should show greater symptom severity, for example; see Figure SF2c.

1. **Exclusion criteria and consenting procedure**

The exclusion criteria for participants in the study included braces, claustrophobia, active substance dependence, pervasive developmental disorder, Tourette’s syndrome, lifetime history of psychosis, neurological disorder, head trauma, non-English speaking, and presence of active safety concerns, non-psychiatric medical illnesses requiring medications that may have psychotropic effects (e.g., beta-blockers, steroids), and IQ<75. Informed consent was obtained from a parent/legal guardian and informed assent was obtained from the youth. In all cases, youth had the right to decline participation at any time before or during the study. It was made clear to all participants and their parents that their decision with respect to participation had no influence on their clinical care.

1. **Measures**

**Clinical measures**

***IQ*** was assessed via the Wechsler Abbreviated Scale of Intelligence II (WASI-II) (perceptual reasoning & verbal comprehension subscales) (Wechsler, 2011). Emotional and behavior problems were indexed via the self-report ***Strengths and Difficulties Questionnaire*** (SDQ) (Goodman, 1997), a 25-item measure with good reliability. Aggression severity was assessed via the ***Reactive-Proactive Aggression Questionnaire (RPAQ; Raine et al., 2006),*** a self-report questionnaire that is a validated measure of both proactive and reactive aggression in youth. ADHD symptoms were measured via the ***Conners 3 ADHD Index*** – Parent (Conners, 2008), a 10-item parent-report scale assessing ADHD symptoms. Depressive symptoms were measured via the ***Children’s Depression Inventory*** (CDI; Kovacs, 2011), a self-report questionnaire that also looks at depressive symptoms in youth and adolescents. Anxiety severity was assessed via the ***Screen for Child Anxiety Related Emotional Disorder*** (SCARED, child version, Birmaher et al., 1997). Prior work has indicated that the SCARED has excellent internal consistency and test-retest reliabilities (Runyon, Chesnut, & Burley, 2018, Stringaris et al., 2012). ***History of maltreatment*** was assessed using the Childhood Trauma Questionnaire (CTQ), a 25-item self-report measure that indexes childhood maltreatment. The CTQ has excellent psychometric properties including internal consistency, test-retest reliability, and convergent and discriminant validity with interviews and clinician reports of maltreatment (Bernstein, Ahluvalia, Pogge, & Handelsman, 1997). Overall alcohol/cannabis consumption over the past year and symptoms of alcohol/cannabis abuse and dependence were assessed via the Alcohol Use Disorder Identification Test (AUDIT) (Bush, Kivlahan, McDonell, Fihn, & Bradley, 1998) and the Cannabis Use Disorder Identification Test (CUDIT) (Adamson & Sellman, 2003). CU traits were assessed via the ***Inventory of Callous-Unemotional Traits*** (ICU(Frick, 2004)). Finally, irritability was assessed via the ***Affective Reactive Index*** (ARI: Stringaris et al., 2012), a seven-item self-report questionnaire with excellent internal consistency and test-retest reliability (Stringaris et al., 2012).

**Passive avoidance learning (PAL) task:** The PAL task, used extensively in previous work with adolescents (Bashford-Largo et al., 2021, White et al., 2013), is a probabilistic instrumental learning paradigm that presents participants with cues that, if acted upon, offer a chance to win or lose virtual money (see Supplemental Figure 1). In each trial, one of four cue shapes was presented. Participants chose whether to respond to the cue. After 1500 ms, the cue was removed, and a fixation cross was presented at a jittered interval (0-4000 ms). If participants chose not to respond to the cue, a blank screen was presented for 1500 ms. If they chose to respond, a feedback screen was presented for 1500 ms informing them that their choice resulted in winning or losing money. Feedback followed a probabilistic reinforcement schedule, wherein: two shapes (high reward probability cues) were associated with an 80% chance of a reward (+$1 or +$5), and a 20% chance of punishment (-$1 or -$5); and two shapes (high punishment probability cues) were associated with an 80% chance of a punishment (-$1 or -$5), and a 20% chance of reward (+$1 or +$5). After choice-feedback or the no-choice blank screen, a second jittered fixation period appeared (0-4000 ms) before a subsequent trial began. Responses were registered via button boxes. Cues were presented in a random order. Each of the four cues were presented 27 times (108 total trials).

1. **fMRI parameters**

Whole-brain blood oxygen level dependent (BOLD) fMRI data were acquired using a 3.0 Tesla Siemens Skyra Magnetic Resonance Scanner. Functional images were taken with a T2* weighted gradient echo planar imaging (EPI) sequence (repetition time [TR] = 2500ms, echo time [TE] = 27ms, flip angle = 90°, field-of-view [FOV] = 240mm). Whole-brain coverage was obtained with 43 axial slices (thickness = 2.5mm; voxel size = 2.6 x 2.6 x 2.5mm^3^). In the same session, a high-resolution T1-weighed anatomical image was acquired to aid spatial normalization (MP-RAGE, TR = 2200ms, TE = 2.48ms; FOV = 230mm; flip angle = 8^o^; 256 x 208 matrix) and registration with the EPI dataset. Whole-brain coverage was obtained with 176 axial slices (thickness = 1mm; voxel size = 0.9 x 0.9 x 1mm^3^).

**fMRI data preprocessing and individual level analysis**

Functional MRI data were preprocessed and analyzed using Analysis of Functional NeuroImages (AFNI) software (Cox, 1996). The first four volumes collected prior to magnetization equilibrium were discarded. The anatomical scan for each participant was registered to the Talairach and Tournoux atlas (Talairach & Tournoux, 1988) and each participant’s functional EPI data were registered to their Talairach anatomical scan using AFNI. Functional images were motion corrected with respect to the initial volume of the first functional run as the reference volume and spatially smoothed with a 6-mm full width at half maximum Gaussian kernel. The data then underwent time series normalization to a T1 image, and these results were multiplied by 100 for each voxel. Therefore, the resultant regression coefficients are representative of a percentage of signal change from the mean.

Following this, regressors depicting each of the response types were created by convolving the train of stimulus events with a gamma-variate haemodynamic response function to account for the slow haemodynamic response. Four regressors were generated: objects chosen, objects refused, reward received, punishment received. Linear regression modelling was then performed using the regressors described above plus regressors to model a fourth order baseline drift function. General Linear Model fitting was performed with these four regressors, six motion regressors, and a regressor modeling baseline drift. All regressors were convolved with a canonical hemodynamic response function (HRF) to account for the slow hemodynamic response (with time point commencing at time of first image onset). This produced a β coefficient and associated *t* statistic for each voxel and regressor. There was no significant regressor collinearity.

***Movement data***

Volumes were censored if there was >0.5 mm motion across adjacent volumes. No participant in the final sample for the current study had >5% censored volumes. There were no significant group differences (TD vs diagnosed adolescents) in terms of censored volumes, average motion per volume or maximum displacement during scanning (F<1 in all cases; n.s.).

1. **Feature creation**

The Schaefer’s Atlas (Schaefer et al., 2018) was used to parcellate the whole brain into 400 regional parcellations. The atlas used in the current study was generated using a stable gradient-weighted Markov Random Field (gwMRF) algorithm that was implemented on structural MRI and resting-state fMRI data from 1489 young adults (Schaefer et al., 2018). This 400 regional parcellation reflects network organization as well (Schaefer et al., 2018).

Subject-wise and hemispheric-wise BOLD response to the receipt of reward and punishment information was determined for each of the 400 regions as well as 12 bilateral subcortical regions (the thalamus, caudate, putamen, nucleus accumbens, hippocampus and amygdala). The whole-brain parcellation into 400 cortical and 12 subcortical regions was performed using the *mri_surf2surf*, *mris_anatomical_stats*, and *aparcstats2table* pipelines following the FreeSurfer *recon‐all* pipeline (Fischl et al., 2002).

***Feature selection and ML analysis***

The data from 69 TD adolescents was used to determine the features selected to make up the hyperplane differentiating the BOLD response to the receipt of reward versus punishment. We took the K_1_ $\times$ K_2_ nested cross-validation approach to estimate model performance (Varma & Simon, 2006), where K_1_ and K_2_ represent the number of outer and inner loops respectively. We chose K_1_ = 10 and K_2_ = 10 in the current study. There is no formal rule to choose the values for K_1_ or K_2_, however, both the 10-fold and the 5-fold cross-validations have been shown empirically to yield test error estimates that maintain a balance between excessively high bias and excessively high variance (please cite this book here: https://vuquangnguyen2016.files.wordpress.com/2018/03/applied-predictive-modeling-max-kuhn-kjell-johnson_1518.pdf). The 10-fold cross-validation is more commonly used (compared to 5-fold cross-validation) just because of better computational efficiency. In the outer loop, the randomized data were split into K_1_ folds where K_1_-1 folds were used as the training data and one-fold was used as the test data. The training data were transformed into z-score, and the corresponding transformations were used to the testing data. The least absolute shrinkage and selection operator (LASSO; Tibshirani, 1996) (function *lasso* from MATLAB R2021a) was used on the training data to select features. This procedure was repeated K_1_ times, each time leading to a set of best features. The final set of features were formed by the features that appeared more than 2 times in the K_1_ sets of best features. The features that were not in this final set were excluded from the training and test data. In the inner loop, the reduced training data were further divided into K_2_ folds i.e., K_2_-1 folds were used as the sub-training data and one-fold was used as the validation data. The inner loop was repeated K_2_ times. In each iteration, the SVM ML classifier was trained using the sub-training data. The obtained model was then tested on the validation data resulting in a value of validation accuracy. The hyperparameters in the model were tuned using Bayesian optimization during each iteration such that the validation accuracy for each model was maximized. The performance of the model which gave the highest validation accuracy from the inner loop was then evaluated on the test data left in the outer loop. This step was repeated K_1_ times. The average of the K_1_ test-accuracies computed from the outer testing folds was regarded as the *generalized accuracy*. The corresponding sensitivity and specificity parameters were regarded as *generalized sensitivity* and *generalized specificity* respectively. All the steps for feature selection and ML analysis were performed in MATLAB R2021a. This ML framework is visually represented in Figure 2 in our previously published work (please cite: <https://link.springer.com/article/10.1007/s44192-023-00033-6>).

The identified features (regions) were then interrogated, via t tests, to determine whether they showed greater BOLD responses to reward or punishment trials.

Following this, distance from the hyperplane was calculated for each participant for both their response to reward and their response to punishment trials. This was calculated not only for the 69 TD participants on the whom the SVM ML was conducted but also the independent sample of 35 TD adolescents and the 195 clinically concerning participants.

1. **Chi-square analysis assessing differences in the proportions of participants in the normed response to reward groups between the patient group and TD_test_ groups**

Chi-square analyses revealed highly significant differences in the proportions of participants in the normed response to reward groups between the patient group and TD_Train_ groups (χ^2^(df[4], N=260) = 29.64, p < 0.001).

1. **Sensitivity analyses:**

***Associations between DFH and clinical data:***

Partial correlations examined associations between DFH for responses to reward and clinical measures indexing conduct problems, hyperactivity and depression across the test sample controlling for age, IQ and sex (see Supplemental Table 5). These largely mirrored the main results (main text: Table 2) except for associations between Reward_DFH_ and both Connors ADHD score and SDQ-Emotional problems.

***Examination of symptom severity as a function of reward response categorization:***

The univariate ANOVAs examining the association of symptom severity as a function of reward response categorization were similarly repeated controlling for age, IQ and sex (see Supplemental Table 6). These largely mirrored the main results (main text: Table 3).

The univariate ANOVAs examining the association of symptom severity as a function of reward response categorization were also repeated excluding participants prescribed anti-psychotic, stimulant or SSRI medication (see Supplemental Table 7). These also largely mirrored the main results (main text: Table 3).

.


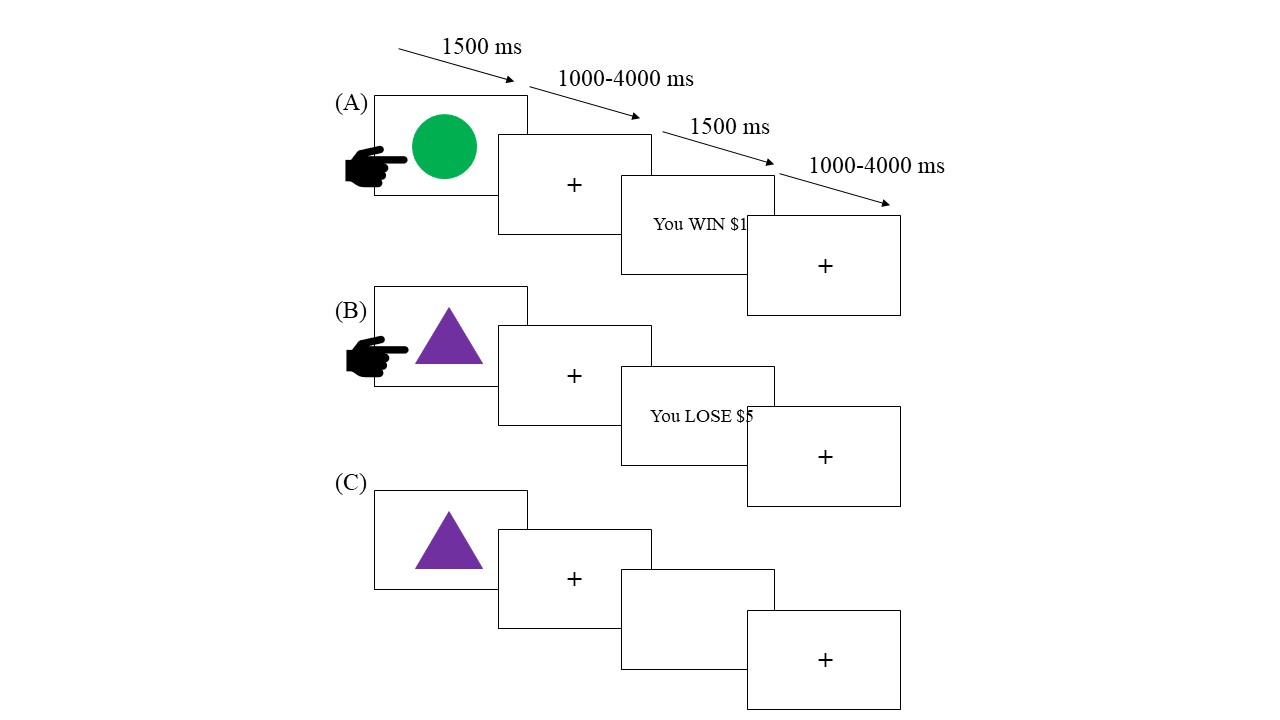
Supplemental Figure 1: Exemplar trials on the passive avoidance task; (A) A trial where the participant respond to the green circle and receives a probabilistic reward; (B) A trial where the participant responds to a purple triangle and receives a probabilistic punishment; and (C) A trial where the participant does not respond and receives neither reward or punishment.

Supplemental Figure 2: 2a: The hyperplane that maximizes the distances from the support vectors corresponding to individual participants’ reward and punishment to the hyperplane. Red dashed two-headed arrow: *Distance from hyperplane (DFH).* Dark blue line: The TD_Train_ mean_DFH_ (the in the schematic Supplemental Figure SF2a). Light blue dashed lines: Two sd_DFH_ +/- the mean_DFH_. Dark orange dot: The support vector of a participant whose reward response distance from the hyperplane is less than 2 TD_Train_ sd_DFH_ from the TD_Train_ mean_DFH_. 2b: Participants as a function of their normed DFH based on the *HC_Train_* data. 2c: Relationship of brain function categories to symptom severity.

**
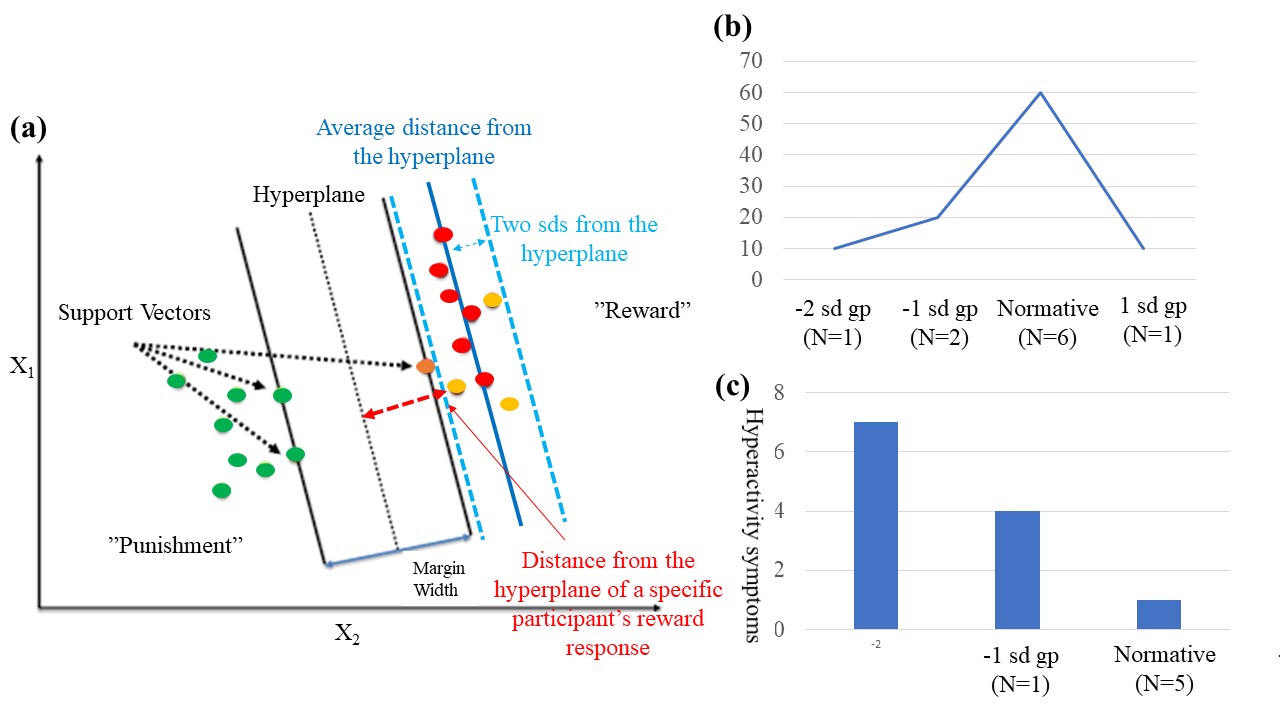
**

**Supplemental Table 1: The number of participants with each comorbidity pattern**

| Diagnoses | N |
| --- | --- |
| ADHD, CD, MDD & GAD | 12 |
| ADHD, CD & MDD | 3 |
| ADHD, CD & GAD | 15 |
| ADHD, MDD & GAD | 7 |
| CD, MDD & GAD | 2 |
| ADHD & CD | 54 |
| ADHD & MDD | 3 |
| ADHD & GAD | 14 |
| CD & MDD | 1 |
| CD & GAD | 4 |
| MDD & GAD | 5 |
| ADHD alone | 32 |
| CD alone | 14 |
| MDD alone | 2 |
| GAD alone | 4 |

Key to Supplemental Table 3: ADHD = Attention Deficit Hyperactivity Disorder; CD = Conduct Disorder; MDD = Major Depressive Disorder; GAD = Generalized Anxiety Disorder.

**Supplemental Table 2: Associations between Distance from the Hyperplane (DFH) and Demographic Data for both TD_Train_ and the test sample (TD_Test_ and participants with a diagnosis)**

|  |  | AGE | IQ |  |  | Sex | |
| --- | --- | --- | --- | --- | --- | --- | --- |
|  |  |  | Correlations | Male | Female | F | p |
| TD_Train_ | Rew | -0.12 | -0.02 | 1.16 | 1.32 | 0.40 | 0.53 |
|  | Pun | 0.04 | -0.02 | -1.47 | -1.61 | 0.19 | 0.66 |
| Test sample TD_Train_ & patients) | Rew | -0.01 | -0.10 | 0.49 | 0.72 | 1.25 | 0.26 |
|  | Pun | -0.07 | **-0.14^*^** | -0.44 | -0.74 | 1.29 | 0.25 |

Key to Supplemental Table 1: Rew: Reward DFH; Pun: Punishment DFH; **^*^** P<0.05

**Supplemental Table 3:** **The number of groups of participants within each normed reward DFH category**

|  | Normed reward DFH categories | | | | |
| --- | --- | --- | --- | --- | --- |
|  | >2 sd_Train_ below average Train_DFH_ | >1 sd_Train_ below average Train_DFH_ | Normative (>1sd_Train_ below and <1sd above average Train_DFH_) | >1 sd_Train_ above average Train_DFH_ | >2 sd_Train_ above average Train_DFH_ |
| TD_Test_ | 3 | 6 | 28 | 1 | 1 |
| Patient | 39 | 50 | 84 | 15 | 7 |
| CD | 22 | 31 | 41 | 8 | 3 |
| ADHD | 24 | 39 | 59 | 13 | 5 |
| GAD | 14 | 18 | 27 | 2 | 2 |
| MDD | 9 | 6 | 18 | 1 | 1 |
| TD_Train_ | 1 | 5 | 50 | 7 | 2 |

Key to Supplemental Table 3: CD = Conduct Disorder; ADHD = Attention Deficit Hyperactivity Disorder; GAD = Generalized Anxiety Disorder; MDD = Major Depressive Disorder.

| **Supplemental Table 4: Associations of Level of Reward response (distance from hyperplane) groups and symptom severity (including those within the increased reward response groups and contrasts of each group against normative response group).** | | | | | | | | | | | | | | | | | | | | | | | | | | | | | |
| --- | --- | --- | --- | --- | --- | --- | --- | --- | --- | --- | --- | --- | --- | --- | --- | --- | --- | --- | --- | --- | --- | --- | --- | --- | --- | --- | --- | --- | --- |
|  | 2 sd below TD M_distance_ from hyperplane | | 1 sd below TD M_distance_ from hyperplane | | | > 1sd below and >1sd above TD M_distance_ from hyperplane | | | 1 sd above TD M_distance_ from hyperplane | | 2 sd above TD M_distance_ from hyperplane | | | | 2sd below vs normative response | | | 1sd below vs normative response | | | 1 sd above vs normative response | | 2 sd above vs. normative response | | |  |  |  |  |
|  |  |  |  |  |  |  |  |  |  |  |  |  |  |  |  |  |  |  |  |  |  |  |  |  |  |  | |  |  |
|  | M | s.d. | M | s.d. | M | | s.d. | M | | s.d. | | M | s.d. | F | | p | F | | p | F | | p | | F | p | |  | |  |
| SDQ-CP | 4.91 | 3.04 | 4.55 | 2.79 | 3.23 | | 3.24 | 3.92 | | 2.72 | | 4.00 | 4.69 | **6.90** | | **0.010** | **5.34** | | **0.022** | 0.54 | | 0.464 | | 0.21 | 0.649 | |  | |  |
| RPAQ-Reactive | 9.89 | 3.98 | 8.62 | 3.64 | 7.26 | | 3.73 | 8.08 | | 3.64 | | 8.50 | 2.12 | **9.61** | | **0.003** | 3.09 | | 0.082 | 0.59 | | 0.446 | | 0.22 | 0.642 | |  | |  |
| RPAQ-Proactive | 5.71 | 3.33 | 5.82 | 3.15 | 5.03 | | 3.04 | 5.08 | | 2.25 | | 4.67 | 1.53 | 0.96 | | 0.331 | 1.52 | | 0.221 | 0.003 | | 0.957 | | 0.04 | 0.839 | |  | |  |
| Conners ADHD | 8.28 | 6.81 | 8.38 | 6.69 | 5.11 | | 5.81 | 9.36 | | 6.85 | | 5.25 | 6.08 | **6.64** | | **0.011** | **7.56** | | **0.007** | **4.97** | | **0.028** | | 0.002 | 0.963 | |  | |  |
| SDQ-Hyperactivity | 6.05 | 3.01 | 5.89 | 3.10 | 4.62 | | 3.33 | 6.85 | | 2.44 | | 4.50 | 4.20 | **5.07** | | **0.026** | **4.41** | | **0.038** | **5.34** | | **0.023** | | 0.005 | 0.944 | |  | |  |
| SDQ-Emotional | 4.42 | 2.86 | 4.12 | 3.30 | 2.85 | | 2.63 | 3.31 | | 2.86 | | 4.00 | 4.90 | **8.59** | | **0.004** | **5.58** | | **0.020** | 0.33 | | 0.567 | | 0.67 | 0.415 | |  | |  |
| CDI | 12.33 | 10.27 | 10.18 | 9.15 | 7.91 | | 6.91 | 9.23 | | 7.72 | | 9.25 | 8.97 | **8.75** | | **0.004** | 2.80 | | 0.096 | 0.41 | | 0.524 | | 0.27 | 0.606 | |  | |  |
| SCARED Total | 18.44 | 15.66 | 20.33 | 16.30 | 17.37 | | 14.78 | 15.57 | | 15.49 | | 17.63 | 13.99 | 0.14 | | 0.706 | 1.30 | | 0.256 | 0.18 | | 0.671 | | 0.002 | 0.963 | |  | |  |
| SCARED GAD | 5.44 | 4.85 | 6.26 | 5.17 | 5.61 | | 4.85 | 4.57 | | 4.56 | | 5.25 | 4.89 | 0.04 | | 0.852 | 0.63 | | 0.429 | 0.57 | | 0.451 | | 0.04 | 0.842 | |  | |  |
| Abuse | 23.76 | 10.49 | 19.46 | 6.07 | 22.11 | | 10.49 | 21.06 | | 16.19 | | 27.25 | 11.00 | 0.76 | | 0.385 | 3.04 | | 0.083 | 0.15 | | 0.702 | | 1.78 | 0.184 | |  | |  |
| Neglect | 19.24 | 9.37 | 15.61 | 5.35 | 15.07 | | 6.12 | 16.19 | | 7.18 | | 14.38 | 3.89 | **10.40** | | **0.002** | 0.31 | | 0.578 | 0.45 | | 0.506 | | 0.10 | 0.752 | |  | |  |
| AUDIT | 3.90 | 5.66 | 2.12 | 3.84 | 1.82 | | 4.57 | 1.40 | | 2.61 | | 7.57 | 12.84 | **4.97** | | **0.027** | 0.15 | | 0.697 | 0.12 | | 0.727 | | **7.34** | **0.008** | |  | |  |
| CUDIT | 7.74 | 9.10 | 7.20 | 9.33 | 4.71 | | 8.01 | 5.87 | | 9.07 | | 7.71 | 11.57 | 3.69 | | 0.057 | 2.87 | | 0.092 | 0.26 | | 0.610 | | 0.86 | 0.355 | |  | |  |
| ICU | 25.40 | 7.85 | 23.38 | 9.12 | 20.58 | | 8.31 | 21.25 | | 8.51 | | 22.86 | 15.72 | **10.60** | | **0.001** | **3.95** | | **0.048** | 0.09 | | 0.763 | | 0.44 | 0.509 | |  | |  |
| ARI | 3.00 | 3.17 | 3.29 | 3.14 | 2.65 | | 2.95 | 2.69 | | 3.09 | | 4.00 | 4.16 | 0.41 | | 0.521 | 1.67 | | 0.198 | 0.002 | | 0.961 | | 1.31 | 0.254 | |  | |  |

Key to Supplemental Table 3: s.d.: standard deviation; TD: Typically Developing; M: Mean; SDQ-CP: Strength and Difficulties Questionnaire – Conduct Problems subscale; RPAS: Reactive Proactive Aggression Scale; SCARED: Screen for Child Anxiety Related Emotional Disorder; AUDIT: Alcohol Use Disorder Identification Test (AUDIT); CUDIT: Cannabis Use Disorder Identification Test; ICU: Inventory of Callous-Unemotional Traits; ARI: Affective Reactive Index

**Supplemental Table 5: Associations between Distance from the Hyperplane and Clinical Data (controlling for age, IQ and sex)**

| **Controlling for:** | **Age** | | **IQ** | | **Sex** | |
| --- | --- | --- | --- | --- | --- | --- |
|  | **Rew** | **Pun** | **Rew** | **Pun** | **Rew** | **Pun** |
| SDQ-CP | -0.19* | 0.01 | -0.21* | -0.03 | -0.19* | 0.01 |
| RPAQ-Reac | -0.23* | -0.02 | -0.25* | -0.06 | -0.23* | -0.02 |
| RPAQ-Proactive | -0.15 | 0.03 | -0.17 | -0.01 | -0.15 | 0.02 |
| Conners ADHD | -0.01 | -0.15 | -0.02 | -0.17 | -0.02 | -0.16 |
| SDQ-Hyperactivity | -0.09 | -0.08 | -0.11 | -0.11 | -0.10 | -0.09 |
| SDQ-Emotional | -0.06 | 0.01 | -0.08 | -0.03 | -0.06 | 0.04 |
| CDI | -0.10 | -0.12 | -0.12 | -0.14 | -0.11 | -0.09 |
| SCARED Total | -0.09 | -0.13 | -0.11 | -0.16 | -0.10 | -0.08 |
| SCARED GAD | -0.03 | -0.08 | -0.05 | -0.10 | -0.04 | -0.04 |
| Abuse | 0.05 | -0.10 | 0.03 | -0.14 | 0.05 | -0.06 |
| Neglect | -0.24* | -0.10 | -0.26* | -0.12 | -0.25* | -0.09 |
| AUDIT | 0.03 | 0.14 | 0.01 | 0.10 | 0.03 | 0.17 |
| CUDIT | -0.09 | 0.10 | -0.11 | 0.05 | -0.09 | 0.10 |
| ICU | -0.15 | -0.05 | -0.17 | -0.08 | -0.16 | -0.04 |
| ARI | -0.10 | -0.03 | -0.12 | -0.07 | -0.10 | -0.01 |

Key to Table 2: SDQ-CP = Strengths and Difficulties Questionnaire – Conduct Problems subscale; RPAS-Reactive = Reactive Proactive Aggression Scale – Reactive subscale; RPAS-Proactive = Reactive Proactive Aggression Scale – Proactive subscale; Conners ADHD: Conners 3 ADHD Index; SDQ-Hyperactivity = Strengths and Difficulties Questionnaire – Hyperactivity subscale; SDQ-Emotional = Strengths and Difficulties Questionnaire – Emotional Problems subscale; CDI = Child Depression Inventory; SCARED Total = Screen for Child Anxiety Related Emotional Disorder Total score; SCARED GAD = Screen for Child Anxiety Related Emotional Disorder GAD subscale; Abuse = Childhood Trauma Questionnaire abuse score (Sexual+Physical+Emotional abuse); Neglect = Childhood Trauma Questionnaire abuse score (Physical+Emotional neglect); AUDIT = Alcohol Use Disorder Identification Test; CUDIT = Cannabis Use Disorder Identification Test; ICU = Inventory of Callous Unemotional Traits; ARI = Affective Reactivity Index;^*^ p<0.05.

**Supplemental Table 6: Significance of Associations of Level of Reward response (distance from hyperplane) groups and symptom severity (TD_Test_ and participants with diagnoses, controlling for age, IQ and sex)**

|  | Age | | IQ | | Sex | |
| --- | --- | --- | --- | --- | --- | --- |
|  | F | p | F | p | F | p |
| SDQ-CP | **5.427** | **0.021** | **12.296** | **<0.001** | **4.706** | **0.004** |
| RPAQ-Reactive | **3.039** | **0.031** | **3.382** | **0.020** | **3.103** | **0.029** |
| RPAQ-Proactive | 0.417 | 0.741 | 0.465 | 0.707 | 0.482 | 0.695 |
| Conners ADHD | **4.245** | **0.007** | **4.083** | **0.008** | **4.197** | **0.007** |
| SDQ-Hyperactivity | **3.074** | **0.029** | **3.118** | **0.028** | **3.392** | **0.019** |
| SDQ-Emotional | **3.444** | **0.018** | **3.670** | **0.014** | **4.252** | **0.006** |
| CDI | 2.571 | 0.056 | 2.615 | 0.053 | **2.688** | **0.048** |
| SCARED Total | 0.562 | 0.641 | 0.578 | 0.630 | 0.687 | 0.561 |
| SCARED GAD | 0.315 | 0.815 | 0.436 | 0.727 | 0.797 | 0.497 |
| Abuse | 2.114 | 0.100 | 2.369 | 0.072 | 1.914 | 0.128 |
| Neglect | **5.488** | **0.001** | **5.675** | **<0.001** | **5.615** | **0.001** |
| AUDIT | 1.290 | 0.279 | 1.215 | 0.306 | 1.375 | 0.252 |
| CUDIT | 0.697 | 0.555 | 0.899 | 0.443 | 1.100 | 0.351 |
| ICU | **3.977** | **0.009** | **4.000** | **0.009** | **4.018** | **0.008** |
| ARI | 0.608 | 0.610 | 0.536 | 0.658 | 0.809 | 0.490 |

**Supplemental Table 7: Significance of Associations of Level of Reward response (distance from hyperplane) groups and symptom severity (TD_Test_ and participants with diagnoses, with participants prescribed anti-psychotic, stimulant or SSRI medications removed)**

|  | Excl. antipsychotic medications | | Excl. stimulant medications | | Excl. SSRI medications | |
| --- | --- | --- | --- | --- | --- | --- |
|  | F | p | F | p | F | p |
| SDQ-CP | **4.609** | **0.011** | **3.502** | **0.033** | **3.822** | **0.024** |
| RPAQ-Reactive | **6.924** | **0.001** | **4.272** | **0.016** | **4.208** | **0.017** |
| RPAQ-Proactive | 1.243 | 0.292 | 0.844 | 0.433 | 0.388 | 0.679 |
| Conners ADHD | **4.183** | **0.017** | 2.071 | 0.130 | **5.544** | **0.005** |
| SDQ-Hyperactivity | **3.633** | **0.029** | 1.792 | 0.171 | **3.348** | **0.038** |
| SDQ-Emotional | **4.079** | **0.019** | **5.125** | **0.007** | **5.021** | **0.008** |
| CDI | **4.742** | **0.010** | **3.948** | **0.021** | **4.470** | **0.013** |
| SCARED Total | 0.573 | 0.565 | 0.369 | 0.692 | 0.712 | 0.492 |
| SCARED GAD | 0.196 | 0.822 | 0.575 | 0.564 | 0.343 | 0.710 |
| Abuse | 2.854 | 0.060 | 2.079 | 0.128 | 1.329 | 0.267 |
| Neglect | **5.187** | **0.006** | **5.110** | **0.007** | **3.051** | **0.050** |
| AUDIT | **3.369** | **0.037** | **4.835** | **0.009** | 1.954 | 0.145 |
| CUDIT | **3.050** | **0.050** | **4.645** | **0.011** | **4.387** | **0.014** |
| ICU | **4.246** | **0.016** | **3.218** | **0.043** | **4.682** | **0.010** |
| ARI | 0.151 | 0.860 | 0.073 | 0.930 | 1.184 | 0.309 |

REFERENCES

Adamson, S. J. & Sellman, J. D. (2003). A prototype screening instrument for cannabis use disorder: the Cannabis Use Disorders Identification Test (CUDIT) in an alcohol-dependent clinical sample. *Drug Alcohol Review,* *22,* 309-15. doi:10.1080/0959523031000154454.

Averbeck, B. & O'Doherty, J. P. (2022). Reinforcement-learning in fronto-striatal circuits. *Neuropsychopharmacology,* *47,* 147-162. doi:10.1038/s41386-021-01108-0.

Bashford-Largo, J., Aloi, J., Zhang, R., Bajaj, S., Carollo, E., Elowsky, J., . . . Blair, K. S. (2021). Reduced neural differentiation of rewards and punishment during passive avoidance learning in adolescents with generalized anxiety disorder. *Depression and Anxiety*. doi:10.1002/da.23150.

Bernstein, D. P., Ahluvalia, T., Pogge, D. & Handelsman, L. (1997). Validity of the Childhood Trauma Questionnaire in an adolescent psychiatric population. *Journal of the American Academy of Child and Adolescent Psychiatry,* *36,* 340-8. doi:10.1097/00004583-199703000-00012.

Birmaher, B., Khetarpal, S., Brent, D., Cully, M., Balach, L., Kaufman, J., . . . Neer, S. M. (1997). The Screen for Child Anxiety Related Emotional Disorders (SCARED): Scale construction and psychometric characteristics. *Journal of the American Academy of Child and Adolescent Psychiatry,* *36,* 545–553.

Bush, K., Kivlahan, D. R., McDonell, M. B., Fihn, S. D. & Bradley, K. A. (1998). The AUDIT alcohol consumption questions (AUDIT-C): an effective brief screening test for problem drinking. Ambulatory Care Quality Improvement Project (ACQUIP). Alcohol Use Disorders Identification Test. *Archives of Internal Medicine,* *158,* 1789-95. doi:10.1001/archinte.158.16.1789.

Clithero, J. A. & Rangel, A. (2014). Informatic parcellation of the network involved in the computation of subjective value. *Social Cognitive and Affective Neuroscience,* *9,* 1289-302. doi:10.1093/scan/nst106.

Conners, C. K. (2008). *Conners' Rating Scales, 3rd Edition*. Multi-Health Systems: North Tonawanda, NY.

Cox, R. W. (1996). AFNI: software for analysis and visualization of functional magnetic resonance neuroimages. *Computers and Biomedical Research,* *29,* 162-73.

Fischl, B., Salat, D. H., Busa, E., Albert, M., Dieterich, M., Haselgrove, C., . . . Dale, A. M. (2002). Whole brain segmentation: automated labeling of neuroanatomical structures in the human brain. *Neuron,* *33,* 341-55.

Frick, P. J. (2004). *Inventory of callous–unemotional traits*. University of New Orleans: New Orleans, LA.

Goodman, R. (1997). The Strengths and Difficulties Questionnaire: a research note. *Journal of Child Psychology and Psychiatry,* *38,* 581-6. doi:10.1111/j.1469-7610.1997.tb01545.x.

Gueguen, M. C. M., Lopez-Persem, A., Billeke, P., Lachaux, J. P., Rheims, S., Kahane, P., . . . Bastin, J. (2021). Anatomical dissociation of intracerebral signals for reward and punishment prediction errors in humans. *Nat Commun,* *12,* 3344. doi:10.1038/s41467-021-23704-w.

Kovacs, M. (2011). *Children’s Depression Inventory 2 (CDI-2)* Multi-Health Systems: Toronto.

Raine, A., Dodge, K., Loeber, R., Gatzke-Kopp, L., Lynam, D., Reynolds, C., . . . Liu, J. (2006). The Reactive-Proactive Aggression Questionnaire: Differential Correlates of Reactive and Proactive Aggression in Adolescent Boys. *Aggressive Behavior,* *32,* 159-171. doi:10.1002/ab.20115.<http://onlinelibrary.wiley.com/store/10.1002/ab.20115/asset/20115_ftp.pdf?v=1&t=hxt61e2m&s=b761422fab119d0683f1b585db87ab8272db3802>

Runyon, K., Chesnut, S. R. & Burley, H. (2018). Screening for childhood anxiety: A meta-analysis of the screen for child anxiety related emotional disorders. *Journal of Affective Disorders,* *240,* 220-229. doi:10.1016/j.jad.2018.07.049.

Schaefer, A., Kong, R., Gordon, E. M., Laumann, T. O., Zuo, X. N., Holmes, A. J., . . . Yeo, B. T. T. (2018). Local-Global Parcellation of the Human Cerebral Cortex from Intrinsic Functional Connectivity MRI. *Cerebral Cortex,* *28,* 3095-3114. doi:10.1093/cercor/bhx179.

Stringaris, A., Goodman, R., Ferdinando, S., Razdan, V., Muhrer, E., Leibenluft, E., . . . Brotman, M. A. (2012). The Affective Reactivity Index: a concise irritability scale for clinical and research settings. *J Child Psychol Psychiatry,* *53,* 1109-17. doi:10.1111/j.1469-7610.2012.02561.x.

Talairach, J. & Tournoux, P. (1988). *Co-planar stereotaxic atlas of the human brain*. Thieme: Stuttgart.

Tibshirani, R. (1996). Regression shrinkage and selection via the lasso. *Journal of the Royal Statistical Society B,* *58,* 267-288.

Varma, S. & Simon, R. (2006). Bias in error estimation when using cross-validation for model selection. *BMC Bioinformatics,* *7,* 91. doi:10.1186/1471-2105-7-91.<https://doi.org/10.1186/1471-2105-7-91>

Wechsler, D. (2011). *Wechsler Abbreviated Scale of Intelligence–Second Edition (WASI-II)*. NCS Pearson: San Antonio, TX.

White, S. F., Pope, K., Sinclair, S., Fowler, K. A., Brislin, S. J., Williams, W. C., . . . Blair, R. J. (2013). Disrupted expected value and prediction error signaling in youths with disruptive behavior disorders during a passive avoidance task. *American Journal of Psychiatry,* *170,* 315-23. doi:10.1176/appi.ajp.2012.12060840.
